# Exercise prescriptions for young people’s emotional wellbeing: a systematic review of physical activity intensity, duration, and modality

**DOI:** 10.3389/fpsyg.2025.1552531

**Published:** 2025-04-02

**Authors:** Wanyu Huang, Tong Lam Wong

**Affiliations:** ^1^School of Education and Social Work, The University of Sydney, Darlington, NSW, Australia; ^2^School of Curriculum Teaching & Inclusive Education, Monash University, Melbourne, VIC, Australia

**Keywords:** physical activity, exercise intensity, exercise duration, exercise modality, mood

## Abstract

**Objective:**

Physical activity (PA) is beneficial to the body and effective in promoting mental health and social relationships, which is one of the most important ways to enhance the quality of life. This review investigated the effects of PA in intensity, duration, and exercise modality on young people’s mood.

**Methods:**

Adhering to PRISMA 2020 guidelines, this study conducted a meticulous search across the Web of Science, PubMed, PsycINFO, and Scopus from May to July 2024, and participants aged between 13 and 28 were specified for inclusion in the study. The search yielded 942 titles and abstracts, and subsequent screening according to the criteria led to the inclusion of 20 studies, with 11 focusing on exercise intensity, four on duration, and five on exercise modality.

**Results:**

Exercise of different intensities and durations had different effects on mood, with moderate intensity having the most significant effect on mood. In terms of duration, exercise lasting 10–30 min was more effective in improving positive mood. In addition, the effects of different modalities of exercise on mood also vary, with current research focusing more on the effects of aerobic exercise on mood.

**Conclusion:**

PA can have a positive impact on mental health that varies by intensity, duration, and modality of exercise; moderate intensity and 30 min of exercise tend to result in the most positive emotions. More research could be conducted in the future in different anaerobic exercises.

## 1 Introduction

The transition from adolescence to adulthood is a crucial stage in the development of college students. Even though these emerging adults aim for high levels of intelligence, ambition, and self-worth (Pedrelli et al., 2015), they still must deal with the usual difficulties of this developmental stage, like finding a place to live, forming relationships, dealing with academic and competitive pressures, dealing with financial stress, and having trouble making critical decisions (Parker et al., 2004). Stress-related mental illnesses, such as anxiety and depression, affect a significant portion of college students and impair their ability to function in everyday life and the classroom (Fam, 2018; Sobocki et al., 2006). People usually experience stress, anxiety, and sadness when confronted with an uncertain or complex circumstance or event (Ketata et al., 2021).

According to the US Department of Health and Human Services (1996), PA is any movement of the body that uses energy from the skeletal muscles and has positive psychological consequences (Miles, 2007). Researchers have extensively researched the preventive effects of PA against stress, anxiety, and depression (Anderson and Shivakumar, 2013; Moljord et al., 2014; Zhang Z. et al., 2022). Numerous age groups and people with or without chronic illnesses have shown that PA has favorable associations with stress, anxiety, and depression (Rethorst et al., 2009; Dinas et al., 2011; Kandola et al., 2019).

A substantial amount of research has been conducted over the last three decades that supports the beneficial effects of exercise on mood, especially its anxiolytic effects. The anxiolytic effect has been supported by several reviews and meta-analyses (e.g., O’Connor et al., 2000; Petruzzello et al., 1991). PA can improve mood, lessen the symptoms of anxiety and depression, and improve mental health in general (Haskell et al., 2007; Lees and Hopkins, 2013). PA is crucial in reducing chronic conditions like obesity, diabetes, and heart disease, according to several studies (Warburton et al., 2006). Frequent PA encourages interpersonal communication and fosters a feeling of community (Bailey et al., 2013). Additionally, frequent exercise is linked to enhanced cognitive abilities, such as memory, focus, and problem-solving skills. Additionally, it reduces the risk of Alzheimer’s disease and cognitive decline (Hillman et al., 2008).

Exercise positively impacts other aspects of mood, such as lowering depression and raising wellbeing (Yeung, 1996; Biddle et al., 2000). There is evidence that moderate exercise improves mood (or helps to maintain mood at a high level). In contrast, strenuous exercise leads to a worsening of mood, and these mood changes are more strongly associated with depression than with anxiety (Peluso and De Andrade, 2005). Negative mood can lead to elevated stress levels, weakening the immune system and increasing susceptibility to various health problems (Pressman and Cohen, 2005). Also, mood affects cognitive functions such as attention, memory, and decision-making (Isen, 2001). Research has shown that the effects of regular PA on mood have been studied primarily through aerobic exercise (Salmon, 2001; Brosse et al., 2002), while anaerobic PA, such as bodybuilding or flexibility training, can also reduce depressive symptoms (Martinsen et al., 1989; Martinsen, 1994).

According to the health-related guidelines of the American College of Sports Medicine (2013), 10 min of exercise throughout the day to achieve the desired 30 min of moderate-intensity exercise. In exploring mood changes, research has typically explored the benefits of 20–40 min of exercise (Glass and Chvala, 2001; Lind et al., 2008; Raedeke, 2007; Berger et al., 2016). Only a few studies have documented improvements in mood after 10, 15, or 20 min of exercise (Rudolph and Butki, 1998; Annesi, 2003). When Hansen et al. (2001) examined how long exercise lasted on mood states, they discovered that 10 min improved confusion, exhaustion, and overall mood disturbance ratings.

Exercise can improve physical functioning, mood, symptom severity, and self-efficacy over at least 12 months (Gowans et al., 2004). The relationship between exercise and mood may be influenced by exercise parameters, i.e., intensity, duration, and modality of exercise (Rocheleau et al., 2004). In this article, we systematically review how these exercise parameters affect mood, contributing to a deeper understanding of the neurophysiological mechanisms of exercise on mood. Understanding how different exercise intensities, durations, and modalities affect mood. Clarifying which exercise parameters are most effective in improving mood can help design more precise and effective exercise interventions.

## 2 Methods

Adhering to PRISMA 2020 guidelines, this study conducted a meticulous search across the Web of Science, PubMed, PsycINFO and Scopus from May to July 2024. The search strategy included terms for: (1) physical activity (“physical exercise OR exercise”); (2) benefits (“advantage OR good”); (3) exercise intensity (“physical exercise intensity OR sports intensity OR strength of intensity OR intensity of exercise”); (4) duration (“period OR time OR time duration OR period duration”); (5) modality (“mode OR sense modality”); (6) young people (“adolescents OR teenager OR young person OR young adult”); (7) mood (“emotion OR emotions OR affect”). Furthermore, the reference lists of all included papers were meticulously examined to uncover any pertinent articles that were not detected in the electronic search. The returned results were screened by the first author to exclude duplicate articles based on titles. The abstracts of the remaining articles were reviewed by a review author.

### 2.1 Inclusion and exclusion criteria

The included studies should meet the following criteria: (1) Studies of young people participants (participants in the 13–28 age range were selected for this paper because there may be some variations in the age range of adolescents and young people across different countries and cultures, but this range from adolescence to early adulthood reflects the impact of PA on various developmental stages and makes the findings more broadly applicable). (2) At least one outcome of mood was recorded both before and after exercising. (3) For the studies of exercise intensity, objective measures of exercise intensity should be used. (4) Original research articles published in peer-reviewed journals. (5) The articles were written in English.

### 2.2 Identification of eligible studies

Collaborative screening was carried out by two authors on Rayyan (a specialized website), so the remaining studies were independently reviewed by different authors against the inclusion and exclusion criteria. At this stage, the Rayyan showed the rate of Aligned is 84%. Then one of author review the conflict, the rate of aligned is 91%, and leave note for another author to discussion. The disagreement was resolved by all the authors according to the online discussion till 99% agreement. Finally download the full text for flowing details criteria.

### 2.3 Data extraction

Data extraction was accomplished by two authors working together. All the literature was imported into Rayyan, and then each study was scrutinized by the two authors individually. The data extracted included the age of the participants, the study design, the study methodology, the measure of PA on the intensity of the exercise, the measure of duration, and the effect of modality. The final stage of the analysis was well aligned between the two authors without any conflict.

### 2.4 Quality assessment

The included studies were rigorously quality assessed for this systematic review using the Cochrane Collaboration Risk of Bias Assessment Tool (Higgins, 2006). The tool systematically assessed the potential bias and methodological quality of each study to ensure that the findings had a high degree of confidence and internal validity. Dissenting studies were finally identified through a discussion between the first and second authors.

## 3 Results

**Figure 1:**
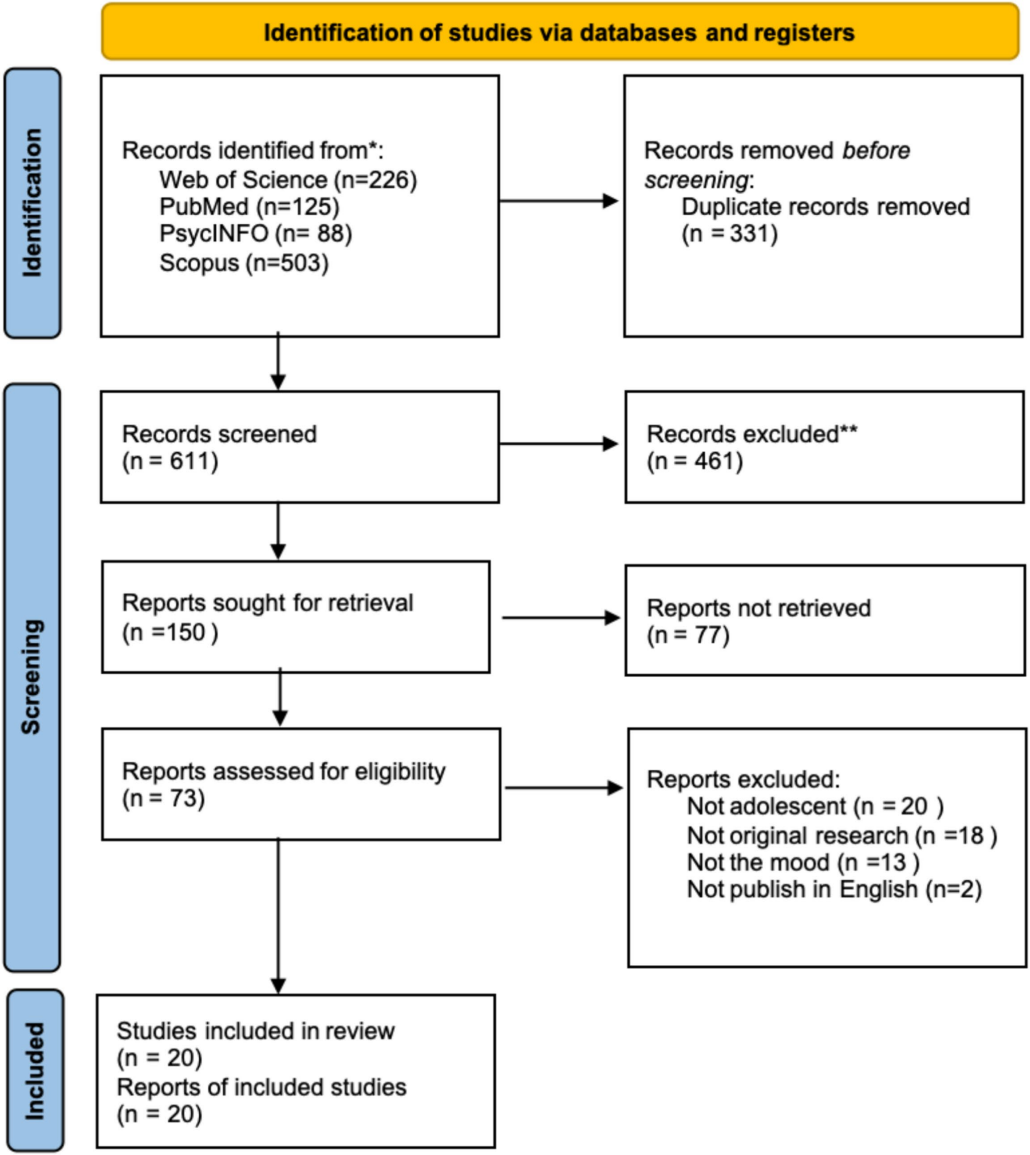
Study selection.

Following PRISMA 2020 guidelines, this study conducted a comprehensive review of 942 papers, and 73 records were retained after the title and abstract screening. Finally, only 20 articles were retained after the full text (see Figure 1).

### 3.1 Risks of bias

**Figure 2:**
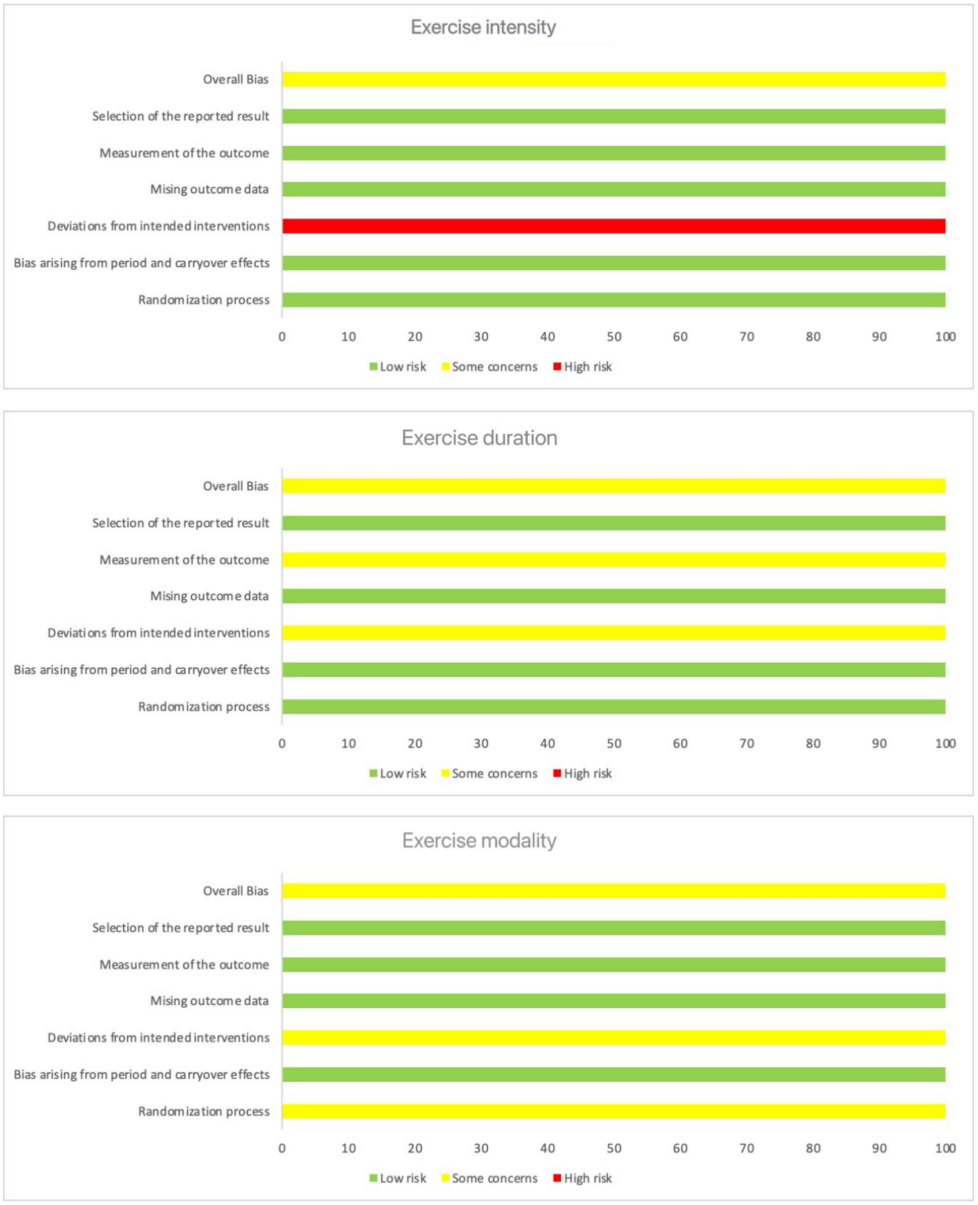
The risk of bias.

This systematic review used the Cochrane Collaboration risk of bias assessment tool to assess the included studies’ quality critically. The tool allowed a comprehensive analysis of each study’s risk of overall biases, selection of the reported result, measurement of the outcome, missing outcome data, deviations from intended interventions, bias arising from period and carryover effects, and randomization process. The assessment results indicated that the results of studies with a low overall risk of bias had a high degree of confidence, although potential limitations in some studies should still be considered with caution (Tables 1–3). Figure 2 summarizes the risk of bias.

**Table 1:** Exercise intensity.

Study	Participants (N)	Sex	Age	Exercise intensity	Findings
Wininger (2007)	College students (*n* = 134)	F/M	\	Riding 20 min on the bike at 70, 50, 30% of estimated VO2max	Changes were most notable for tense arousal negative (i.e., anxiety) for those assigned to the low and moderate intensity exercise conditions.
Kilpatrick et al. (2024)	Young adults (*n* = 27)	F/M	Age = 23	Ramp protocol was performed on an electronically braked cycle ergometer moderate and vigorous intensities (i.e., 20 min below VT and 10 min above VT)	Exercise intensity affects affective responses during exercise.
Morgan et al. (1988)	College swimmers (*n* = 12)	M	\	Daily training distance was increased from 4,000 to 9,000 m.d-1 intensity was maintained at 94% of VO2max	Ratings of exercise intensity, muscle soreness, depression, anger, fatigue, and overall mood disorders increased significantly, while overall wellbeing decreased.
Zhang Q. et al. (2022)	College students (*n* = 739)	F/M	Age = 20	Lower and higher Exercise	High and low exercise intensity had different effects on the relationship between self- concept and negative emotion.
Zhang Z. et al. (2022)	College students (*n* = 1,117)	F/M	Age = 19	Bicycle ergometer	PA reduces negative emotions.
Naugle et al. (2014)	College students (*n* = 22)	\	Age = 20	Low-intensity (treadmill walking, stationary cycling) High-intensity (4 Nintendo Wii games: boxing, tennis, cycling, aerobic step)	Ratings of enjoyment and the increase in positive emotion were greater for boxing and for tennis compared with those for traditional exercises.
Schmitt et al. (2019)	Athletes (*n* = 22)	M	Age = 27	Low (35% below lactate threshold) intensity High (20% above lactate threshold) intensity	There was a significant increase in positive mood in both exercise conditions.
Laki et al. (2022)	Young athletes (*n* = 18)	M	Age = 13	Treadmill (moderate-intensity exercise, 60% MHR)	There was no change in 10 (more than half) adolescent participants, with one child reporting increased affective valence.
Yang et al. (2022)	College students (*n* = 586)	F/M	Age = 20	Low, moderate and high PA	PA could not only directly affect the depressive symptoms of college students but also indirectly affect depressive symptoms through the mediating effect of perceived stress and psychological procrastination.
Seo (2023)	Healthy men (*n* = 9)	M	Age = 25	Cycling exercise consists of three different work roads at 50, 100, and 100 watts for 10 min each	After maximal cycling exercise, total mood disorders did not improve.
Yue and Xiao (2022)	College students (*n* = 100)	F/M	\	Aerobic exercise 30 min	Moderate-intensity exercise induces a happy emotional state in the subjects.

F, Female; M, Male.

**Table 2:** Exercise duration.

Study	Participants (N)	Sex	Age	Exercise duration	Findings
Daley and Welch (2004)	College students (*n* = 23)	F/M	Aged = 22	15 min and 30 min cycle ergometer exercise	15 and 30 min of moderate-intensity exercise will result in a positive emotional response.
Rudolph and Butki (1998)	College students (*n* = 36)	F	\	10, 15, and 20 min of treadmill running	10 min of aerobic exercise is sufficient to enhance exercise-related self-efficacy and affect.
Berger et al. (2016)	College students (*n* = 55)	F/M	Age = 20	15 min Jogging	College students can experience acute mood benefits after only 15 min of jogging at their preferred intensities.
Smith et al. (2002)	College students (*n* = 24)	F	Age = 22	25 min of low- and moderate-intensity cycling	Anxiety-reducing conditions of cycling exercise or seated rest do not alter emotional responsiveness in healthy college women.

F, Female; M, Male.

**Table 3:** Exercise modality.

Study	Participants (N)	Sex	Age	Exercise modality	Findings
Stenling et al. (2024)	College students (*n* = 52)	F/M	Age = 20	Stair climbing	Compared to a no-exercise control session, following the stair climbing participants exhibited superior cognitive switching performance and reported feeling more energetic and happier.
Miller et al. (2005)	College students (*n* = 34)	F	Age = 21	20 min of exercise on (stair stepper, treadmill, rower, stationary cycle ergometer, and simulated cross-country skiing) Intensity: 65–75% of HRR	Mode preference moderated the improvement in positive affect, with no effect on the reduction in negative affect.
Legrand et al. (2022)	College students (*n* = 150)	F/M	age = 20	Green Walking, Urban Walking and No-exercise	Green walking exercise significantly improved positive affect and significantly reduced negative affect.
Wang et al. (2023)	College students (*n* = 55)	F/M	Age = 20	Tai Chi	Tai chi exercise improves emotional regulation.
Aoki et al. (2017)	Volleyball players (*n* = 18)	M	U16 (Age = 15) U19 (Age = 17)	Volleyball training	U16 group presented a higher value for the total mood disturbance and for the subscales, tension, depression, anger, and fatigue.

F, Female; M, Male.

### 3.2 Intensity of exercise

Exercise intensity is based on the level of energy expenditure, categorizing activity using low, medium, and high levels, and is specifically described as heart rate and metabolic equivalents (Haskell et al., 2007). Most studies have explored the effects of exercise intensity on mood, totalling 11 related papers. PA usually improves mood, but the impact varies across different intensities. Research has shown that it can effectively enhance an individual’s psychological resilience and reduce negative emotions, especially in the case of low-intensity exercise (Zhang Q. et al., 2022). However, high-intensity and low-intensity exercise show different effects on the relationship between self-concept and negative affect. High-intensity training may increase depression, anger, and fatigue in athletes (Morgan et al., 1988), whereas mood dysregulation did not improve after extreme cycling (Seo, 2023).

Low- and moderate-intensity exercise elevates positive mood and improves anxiety (Wininger, 2007; Schmitt et al., 2019; Yue and Xiao, 2022). In contrast, moderate-intensity strength training has the best anxiety-relieving and vigor-enhancing effects in young adults (Arent et al., 2005; O’Connor et al., 1993). High-intensity exercise, such as boxing and tennis, resulted in significantly higher pleasure and positive mood increases than low-intensity exercise (Naugle et al., 2014). Research has indicated that PA improves depression in college students regardless of training intensity (Yang et al., 2022), and typically their negative mood decreases while their positive mood stays the same (Bixby et al., 2001).

### 3.3 Duration of exercise

Exercise duration is the length of an exercise activity and can vary depending on the goal (Garber et al., 2011). Exercise is a proven enhancer of mood among young adults, with a broad range of studies confirming that different durations and types of PA, including cycling and treadmill running, lead to marked improvements in psychological distress, fatigue, and overall mood. Short-duration (10–20 min) runs have been shown to increase positive wellbeing and reduce psychological distress (Rudolph and Butki, 1998; Berger et al., 2016), while moderate-intensity exercise (15–30 min) also produces positive emotional responses, and these effects persist over time (Daley and Welch, 2004). However, there is also a study that suggests that anxiety-reducing conditions of cycling exercise or seated rest do not alter emotional responsiveness in healthy college women (Smith et al., 2002). Anxiety-reducing conditions of cycling exercise or seated rest do not change emotional responsiveness in healthy college women.

### 3.4 Modality of exercise

Exercise modalities refer to specific categories of PA and are often categorized according to exercise intensity, target muscle groups, and physical demands (Caspersen et al., 1985). This evidence emphasizes the significant psychological benefits of PA for young people and calls for integrating exercise into educational and recreational activities to promote mental health and emotional resilience. For example, Stenling et al. (2024) found that participants who climbed stairs reported feeling more energized and happier. Modality preference moderated improvements in participants’ positive mood but had no significant effect on reductions in negative mood (Miller et al., 2005). Legrand et al. (2022) showed that green walking significantly increased participants’ positive and reduced negative moods. In contrast, the same exercise in an urbanized area only effectively reduced negative mood. Wang et al.'s (2023) study also demonstrated that Tai Chi had an improving effect on mood.

## 4 Discussion

Research reveals that low- to moderate-intensity exercise is beneficial for mood improvement and psychological resilience, often reducing negative emotions such as anxiety and stress (Zhang Z. et al., 2022; Wininger, 2007). Studies by Schmitt et al. (2019) and Yue and Xiao (2022) align with these findings, indicating that lower-intensity activities are effective in managing anxiety and enhancing positive moods. However, while this body of evidence supports the efficacy of low-intensity exercise for emotional wellbeing, the benefits might not be as uniform or enduring for all populations. For instance, certain groups, like highly stressed individuals or those with existing mood disorders, may require more targeted interventions beyond general low-intensity exercise routines to achieve sustained emotional benefits. While it may increase vitality, it can also heighten anxiety in some individuals (Ruiz-Ariza et al., 2017), pointing to an ambivalent relationship between high-intensity activities and mood. Morgan et al. (1988) indicate that intense PA can increase depression, anger, and fatigue, potentially due to the physical and psychological stress imposed on the body. This raises questions about whether high-intensity exercise could be unsuitable for individuals already prone to negative emotions, as its demanding nature might exacerbate rather than relieve mental strain.

Interestingly, other studies provide contradictory findings on high-intensity exercise’s mood effects. Naugle et al. (2014) report that sports like boxing and tennis, often involving higher intensities, correlate with increased pleasure and positive mood boosts. These findings suggest that enjoyment of the activity itself and the social or motivational context may play significant roles in determining emotional outcomes from exercise. This potential influence of personal preference or engagement is a notable factor, yet it is often overlooked in studies that solely focus on physical intensity levels. Moderate-intensity exercise may strike the most balanced approach, delivering mood-enhancing benefits without the risks of emotional strain associated with high-intensity exercise. Arent et al. (2005) and O’Connor et al. (1993) find that moderate-intensity strength training is particularly effective for reducing anxiety and enhancing vigor in young adults. These findings imply that moderate-intensity activities may be most suitable for general populations looking to improve their mood and mental wellbeing. However, this “middle ground” effect may vary depending on factors like age and fitness level, as Laki et al. (2022) indicate that moderate-intensity activities have limited effects on adolescent mood. This disparity suggests that mood responses to moderate-intensity exercise might require age or developmental consideration to optimize its effectiveness for emotional health. One limitation across these studies is the variability in measurement tools and sample sizes, which can affect the reliability of their findings. Many studies use self-reported mood measures, which, while valuable, introduce subjective biases. Future research could benefit from more objective mood assessments and larger, more diverse samples to enhance generalizability. Additionally, the long-term effects of consistent exercise intensity on mood are relatively understudied, with most research focusing on short-term impacts. Understanding the sustained effects of different exercise intensities on mood could help clarify whether certain intensities yield lasting benefits or if they require continuous engagement to maintain positive emotional outcomes.

The evidence supporting exercise as an enhancer of mood among young adults is substantial, with numerous studies confirming that both the modality and duration of PA play a significant role in influencing psychological outcomes. The general consensus is that exercise, whether cycling, running, or moderate-intensity activities, tends to improve psychological distress, fatigue, and overall mood. Studies such as Rudolph and Butki (1998) and Berger et al. (2016) have shown that short-duration runs (10–20 min) can effectively reduce psychological distress and boost positive wellbeing. Similarly, moderate-intensity exercise lasting between 15 and 30 min produces lasting emotional benefits, as demonstrated by Daley and Welch (2004), with improvements that persist beyond the immediate exercise period. However, some studies have presented contradictory findings, particularly regarding the effects of exercise on emotional responsiveness in certain populations. For instance, Smith et al. (2002) reported that anxiety-reducing conditions such as cycling exercise or seated rest did not alter emotional responsiveness in healthy college women. This raises an important issue about individual variability in how different exercise types and conditions affect emotional states. Factors such as baseline mood, fitness level, and even the environment in which the exercise takes place could influence the outcomes, suggesting that generalized conclusions about exercise’s emotional benefits might overlook the complexity of these variables.

Duration also appears to be a key factor in determining the psychological benefits of exercise. Shorter sessions, ranging from 15 to 16 min, have been shown to have a quick impact on mood and self-efficacy (Ruiz-Ariza et al., 2019; McDonough et al., 2021), highlighting the rapid emotional benefits of brief, consistent exercise bouts. However, the question remains whether these improvements are sustainable or merely momentary boosts in mood. While moderate-duration exercise (30–45 min) has consistently been linked to mood enhancement (‌Bakir and Kangalgil, 2017; Stolarska et al., 2019), longer exercise sessions (60–90 min) are thought to provide more sustained benefits (Roh et al., 2018). This suggests that while short sessions might be ideal for quickly alleviating distress, longer sessions are necessary for more sustained mood improvements. One critical point to consider is the diversity in individual responses to exercise duration and intensity. For example, some studies suggest that longer sessions may lead to fatigue or negative mood changes, especially if the intensity is too high or the individual is not well-conditioned (Fidelix et al., 2019). This highlights the importance of tailoring exercise routines to individual needs and ensuring that the intensity and duration are appropriate to the person’s fitness level and emotional state.

Research by Ruiz-Ariza et al. (2017) and Herbert et al. (2020) highlights that high-intensity interval training (HIIT) can significantly improve mood states, potentially boosting emotional intelligence and resilience. Similarly, studies by Brand et al. (2019) and Fidelix et al. (2019) point to the benefits of extended aerobic exercises, such as running or cycling, in fostering mood enhancement and boosting self-esteem. These findings align with the general understanding that regular PA plays a crucial role in mental health improvement. Moreover, ‌Bakir and Kangalgil (2017) supports this notion, demonstrating that organized team sports and social physical activities not only improve physical health but also have profound effects on mental health. These activities foster social interaction and a sense of community, which can improve emotional resilience and reduce feelings of isolation. Furthermore, Roh et al. (2018) and Znazen et al. (2021) found that specialized training—such as taekwondo and strength training—can lead to significant improvements in mood, further underscoring the importance of targeted physical activities in mental health promotion.

However, while the general benefits of exercise on mood are well documented, the type, intensity, and duration of the PA can significantly influence the psychological outcomes. For instance, Stenling et al. (2024) demonstrated that even brief activities, such as stair climbing, could increase feelings of energy and happiness, highlighting the impact of even short bursts of PA. This suggests that mood improvements do not necessarily require long durations or high-intensity efforts. On the other hand, Miller et al. (2005) observed that while modality preference (the choice of exercise) influenced participants’ positive mood improvements, it had no significant effect on reducing negative emotions. This indicates that while enjoyment and preference are essential for sustained engagement, they might not directly correlate with reductions in negative mood states. Additionally, the environment in which exercise takes place plays a pivotal role in its emotional outcomes. Legrand et al. (2022) showed that green walking—conducted in natural environments—significantly boosted participants’ positive moods and reduced negative emotions. In contrast, the same exercise performed in urban settings only led to reductions in negative mood, suggesting that the physical environment contributes to the psychological benefits of PA. This highlights the growing body of evidence suggesting that environmental factors, such as green spaces, are essential in optimizing the mood-enhancing effects of exercise. Furthermore, Wang et al.’s (2023) study on Tai Chi illustrates the significance of specific exercises in mood improvement. Tai Chi, a form of mind–body practice, has been shown to improve mood in a more controlled, mindful manner, pointing to the broader spectrum of exercise types that cater to different emotional needs.

Critically, while these findings are promising, they also raise questions about the consistency of exercise’s emotional benefits across different populations. Factors such as baseline mood, individual preferences, and cultural context could influence how young people respond to various forms of exercise. Additionally, the duration and intensity of PA that maximally benefits emotional health may vary, as evidenced by studies that suggest moderate or low-intensity exercises may be more effective in some populations, particularly those with mental health concerns. Thus, future research should aim to explore these variables further, assessing how individualized exercise prescriptions can best address mood disturbances in young people.

## 5 Limitation

Current research has focused on university student populations and the effects of aerobic exercise on mood and mental health, and whilst these studies have shown that aerobic exercise can significantly improve mood states, such as alleviating anxiety and depression, the potential benefits of anaerobic exercise are equally worthy of in-depth exploration. Strength training and high-intensity interval training (HIIT) have been shown to play an important role in elevating mood and increasing mental toughness. Studies have shown that strength training can increase self-esteem, improve emotional stability, and reduce stress levels, while HIIT has received increasing attention for its short duration and efficiency. However, the majority of participants in these studies were university students, and the limitations of the sample prevented the findings from being generalized to a wider group of young people. Differing cultural backgrounds, socioeconomic status, and external factors such as family support, social environment, and academic pressures may significantly impact mood improvement with exercise, and these variables have not been adequately considered in the existing literature. In addition, existing studies vary in their definitions and measurements of exercise intensity, duration, and modality of exercise, further limiting the comparability and generalizability of findings.

Particularly in the field of anaerobic exercise, there is less current research addressing specific mechanisms of mood regulation. For example, there are no consistent findings on whether strength training improves mood through neuroendocrine changes or other physiological mechanisms. There is also a lack of systematic research on how different intensities of anaerobic exercise affect mental health and whether there is an optimal intensity for individual needs. Whether team-based forms of resistance training provide additional mood benefits or whether they improve engagement has also not been explored in depth. Therefore, future studies should be more comprehensive and diverse in their methodological design, expand the sample coverage to include people of different age groups, cultural backgrounds, and health conditions, and focus on the specific pathways and mechanisms of action of anaerobic exercise in improving mental health. In addition, there is a need to further explore the personalized application of anaerobic exercise, such as optimizing the intensity, frequency, and duration of exercise to meet the psychological needs of different individuals. By incorporating the combined effects of anaerobic and aerobic exercise into the study, it can provide an important basis for the development of a more comprehensive and scientific exercise intervention strategy and ultimately a more comprehensive understanding of the multidimensional role of PA in mental health improvement.

## 6 Conclusion

PA has significant benefits on young people’s mood, and its effects are influenced by the intensity, duration, and modality of exercise. Low-intensity exercises helps reduce negative emotions and increase mental toughness; moderate-intensity exercises effectively reduces anxiety and increases vitality, and high-intensity exercises may result in positive or negative emotions. Short periods of running can enhance wellbeing, and 15–30 min of exercise can lead to lasting positive emotions. The positive effects of PA on mood need to be translated into school, family, and government interventions to help youth improve their mood through PA. It is recommended that schools schedule 10–30 min of moderate-intensity exercises daily, such as running or jumping rope during school hours, to improve concentration and reduce stress. At the same time, diversified choices of exercises and mood regulation courses should be provided. Parents can draw up exercises to encourage their children to be less sedentary, e.g., 10–15 min of exercises after using electronic devices. Secondly, the government should publicize the benefits of exercises on mental health through the media to raise public awareness of the importance of exercises for physical health and emotional improvement.

## Data Availability

The original contributions presented in the study are included in the article/supplementary material, further inquiries can be directed to the corresponding author.
